# P-1621. Effectiveness of Mobile Vaccine Clinics on COVID-19 Vaccination Uptake in the United States: Real-world Outcomes Study

**DOI:** 10.1093/ofid/ofaf695.1798

**Published:** 2026-01-11

**Authors:** Khanh Duong, Yue Zhang, Richard Nelson, Andrew T Pavia, Barbara E Jones, Danielle Nguyen, Cindy Wynette, Makoto M Jones, Matthew H Samore, Nathorn Chaiyakunapruk

**Affiliations:** University of Utah, Salt Lake City, Utah; University of Utah, Salt Lake City, Utah; University of Utah, Salt Lake City, Utah; University of Utah, Salt Lake City, Utah; University of Utah and Salt Lake City VA Healthcare System, Salt Lake City, Utah; University of Utah, Salt Lake City, Utah; Utah Department of Health and Human Services, Salt Lake, Utah; Veterans Affairs, Salt Lake City, Utah; University of Utah, Salt Lake City, Utah; University of Utah, Salt Lake City, Utah

## Abstract

**Background:**

Mobile vaccine clinics (MVCs) strategy has been shown to increase COVID-19 vaccine uptake in several countries. However, their impact on overall vaccination rates remains limited in the United States (US). This study aimed to evaluate the effectiveness of MVCs in increasing COVID-19 vaccination uptake in the state of Utah.

**Figure:**
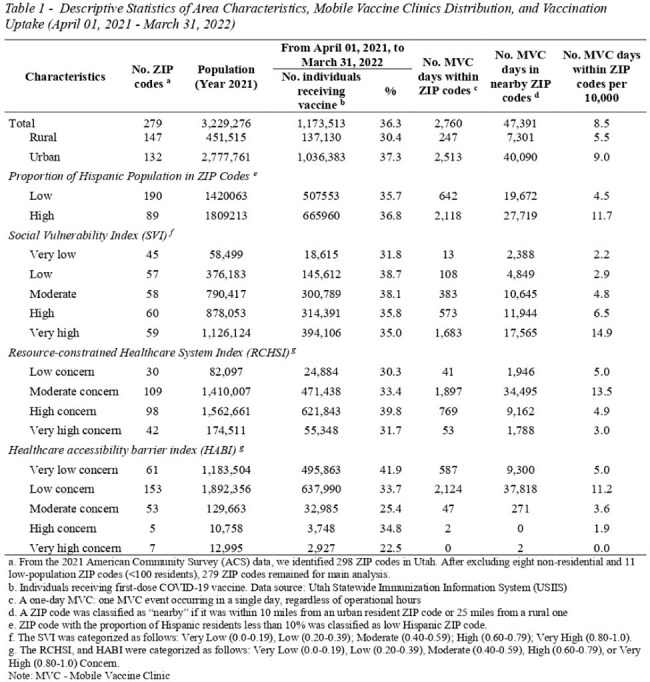

**Figure:**
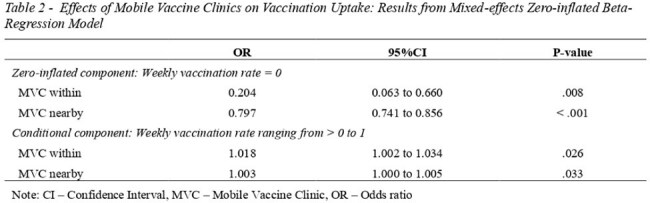

**Methods:**

We collected and analyzed data on MVCs and COVID-19 vaccinations in Utah from April 1, 2021, to March 31, 2022. The primary exposure was the weekly number of MVC days in host ZIP codes. The secondary exposure was the weekly number of MVC days in nearby ZIP codes. The outcome was the weekly first-dose COVID-19 vaccination rate (weekly number of individuals receiving first-dose vaccine/ ZIP code population). We used a mixed-effect zero-inflated beta-regression model to measure the association between a one-day MVC and this outcome. Covariates included healthcare access barriers index, resource-constrained healthcare systems index, social vulnerability index (SVI), vaccine hesitancy, and baseline vaccination rates. The unit of analysis was the ZIP code-week.

**Figure:**
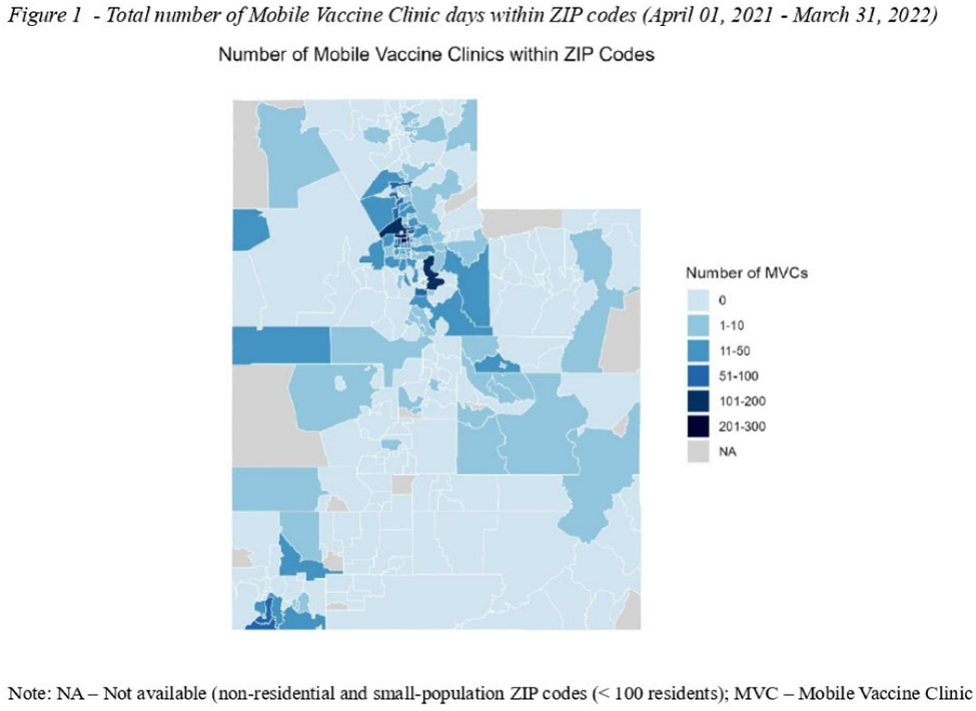

**Results:**

During the study period, MVCs were deployed in Utah for 2,760 MVC days, averaging 8.5 MVC days per 10,000 residents. MVCs were deployed with high density in ZIP codes with high proportions of Hispanics (11.7 days per 10,000 residents), urban ZIP codes (9.0 days per 10,0000 residents), and ZIP codes with very high and high SVI (14.9 and 6.5 days per 10,000 residents, respectively). In the zero-inflated component, each additional MVC day in a given week reduced the odds of no vaccination in that week by 79.6% in host ZIP codes (OR= 0.204, 95%CI 0.063; 0.660), and by 20.3% in nearby ZIP codes (OR= 0.797, 95%CI 0.741; 0.856). In the beta component, for ZIP codes with vaccinations, each additional MVC day in a given week increased the odds of weekly vaccination rates by 1.8% in host ZIP codes (OR= 1.018, 95%CI 1.002; 1.034) , and by 0.3% in nearby ZIP codes (OR= 1.003, 95%CI 1.000; 1.005).

**Conclusion:**

MVCs increased COVID-19 vaccination uptake on both host and nearby ZIP codes in Utah. This finding supports the use of MVCs to improve vaccine coverage and inform their use for other public health programs.

**Disclosures:**

Andrew T. Pavia, MD, Antimicrobial therapy inc.: Royalties|Haleon: Advisor/Consultant

